# The economic burden of inadequate consumption of vegetables and fruit in Canada

**DOI:** 10.1017/S1368980016002846

**Published:** 2016-11-07

**Authors:** John Paul Ekwaru, Arto Ohinmaa, Sarah Loehr, Solmaz Setayeshgar, Nguyen Xuan Thanh, Paul J Veugelers

**Affiliations:** 1 School of Public Health, University of Alberta, 3–50 University Terrace, 8303 – 112 Street, Edmonton, Alberta, Canada, T6G 2T4; 2 Institute of Health Economics, Edmonton, Alberta, Canada

**Keywords:** Economic burden of disease, Nutrition, Public health, Health-care costs, Vegetables and fruit

## Abstract

**Objective:**

Public health decision makers not only consider health benefits but also economic implications when articulating and issuing lifestyle recommendations. Whereas various estimates exist for the economic burden of physical inactivity, excess body weight and smoking, estimates of the economic burden associated with our diet are rare. In the present study, we estimated the economic burden attributable to the inadequate consumption of vegetables and fruit in Canada.

**Design:**

We accessed the Canadian Community Health Survey to assess the inadequacy in the consumption of vegetables and fruit and published meta-analyses to assemble risk estimates for chronic diseases. Based on these inadequacy and risk estimates, we calculated the population-attributable fraction and avoidable direct and indirect costs to society. Direct costs include those for hospital care, physician services and drugs in 2015.

**Results:**

About 80 % of women and 89 % of men consume inadequate amounts of vegetables and fruit. We estimated this to result in an economic burden of $CAN 3·3 billion per year, of which 30·5 % is direct health-care costs and 69·5 % is indirect costs due to productivity losses. A modest 1 percentage point annual reduction in the prevalence of inadequate vegetables and fruit consumption over the next 20 years would avoid approximately $CAN 10·8 billion, and an increase of one serving of vegetables and fruit per day would avoid approximately $CAN 9·2 billion.

**Conclusions:**

Further investments in the promotion of vegetables and fruit will prevent chronic disease and substantially reduce direct and indirect health-care costs.

Chronic diseases are the leading cause of morbidity and mortality worldwide^(^ ^1^ ^)^. In Canada, chronic diseases account for 89 % of all deaths^(^ ^2^ ^)^ and for more than $CAN 93 billion in direct and indirect health-care costs per year^(^ ^3^ ^)^. Dietary intake, sedentary lifestyle, body weight and cigarette smoking have long been established as modifiable risk factors for chronic disease^(^ ^4^ ^)^. Despite the issuing of public health recommendations to reduce the burden of chronic diseases, recent studies revealed that 74 % of Canadians consume less than the recommended daily number of servings of vegetables and fruit^(^ ^5^ ^)^, 85 % of adults are not meeting recommendations for physical activity^(^ ^6^ ^)^, 62 % have excess body weight^(^ ^7^ ^)^ and 18·1 % use tobacco^(^ ^7^ ^)^.

Public health decision makers not only consider potential health benefits but also financial implications when articulating and issuing their recommendations. Various studies have estimated the economic burden in terms of costs for treatment and management of chronic diseases and for productively losses resulting from physical inactivity, excess body weight and smoking^(^ ^8^ ^–^ ^16^ ^)^. For Canada, Krueger *et al*. estimated the economic burden attributable to physical inactivity, excess body weight and tobacco smoking to be $CAN 10·8 billion, $CAN 23·3 billion and $CAN 18·7 billion per year, respectively^(^ ^17^ ^)^. Estimates of the economic burden associated with dietary intake, however, are uncommon. For Australia, the economic burden attributable to low vegetables and fruit consumption was estimated to be $AU 269 million in 2008^(^ ^15^ ^)^, and for the UK the costs for treatment and management of chronic diseases attributable to a poor diet to be £4·9 billion in 2006/2007^(^ ^15^ ^)^. No such estimates exist for the Canadian setting, whereas country-specific estimates are essential to reflect differences in lifestyle, culture, food choices, dietary recommendations and health-care costs. Since the majority of Canadians are not meeting the recommendations for vegetables and fruit^(^ ^5^ ^)^, the associated economic burden is expectedly substantial. An estimate of this burden may help prioritizing public health intervention strategies. The objective of the present study was to estimate the economic burden attributable to the inadequate consumption of vegetables and fruit in Canada.

## Methods

We estimated the economic burden associated with inadequate consumption of vegetables and fruit with a population-attributable fraction (PAF) approach and a societal perspective^(^ ^18^ ^)^. In doing so, we considered the consumption of vegetables and fruit by Canadians, the risk that inadequate consumption poses for chronic diseases and the costs for the treatment and management of these diseases, as detailed below.

### Consumption of vegetables and fruit in Canada

The Canadian Community Health Survey (CCHS) is a large cross-sectional survey that collects information related to health status, health care and health determinants for the Canadian population^(^ ^19^ ^)^. The CCHS collects data from participants 12 years of age or older living in the ten provinces and the three territories of Canada and incorporates population weighting to provide estimates that are representative for the Canadian population. Since 2007, data for the CCHS are collected annually instead of every two years with a target sample size of 65 000 respondents each year. We accessed the 2014 responses of 63 964 Canadians to validated questions on the consumption frequency of vegetables and fruit. These responses had been summarized by means of a single variable representing the total number of servings of vegetables and fruit consumed daily. Since risk estimates are available for increases per 80 g (1 serving) per day in vegetables and fruit intake (see next section), we retrieved age- and gender-specific distributions of vegetables and fruit consumption in the intervals of 0–<1, 1–<2, 2–<3, 3–<4, 4–<5, 5–<6, 6–<7, 7–<8 and 8+ servings/d. Since the CCHS covers only participants aged 12 years or older, we applied the distribution of vegetables and fruit consumption observed in the 12–14 years age group to those under 12 years old. In Canada, males are recommended a minimum of 4–6, 8, 8–10 and 7 servings vegetables and fruit/d for the age groups 2–<14, 14–<19, 19–<51 and 51+ years, respectively. For females, this is a minimum of 4–6, 7, 7–8 and 7 servings vegetables and fruit/d for the age groups 2–<14, 14–<19, 19–<51 and 51+ years, respectively. To align these age-specific recommendations with the available age categories for health-care costs (see below), we adapted this for males to a minimum of 5, 8 and 7 servings vegetables and fruit/d for age groups <15, 15–<55 and 55+ years, respectively. For females, we considered a minimum of 5 and 7 servings vegetables and fruit/d for age groups <15 and 15+ years, respectively.

### Risk of inadequate vegetables and fruit consumption for chronic disease

We accessed reports by the WHO and the World Cancer Research Fund/American Institute for Cancer Research, complemented with published meta-analyses, to assemble established estimates of the risk of inadequate vegetables and fruit consumption for chronic diseases^(^ ^4^ ^,^ ^20^ ^–^ ^24^ ^)^. Based on these sources, we considered risk estimates for type 2 diabetes^(^ ^21^ ^)^, CVD (IHD, ischaemic stroke)^(^ ^4^ ^)^ and cancers (lung^(^ ^22^ ^)^, colorectal^(^ ^25^ ^)^, oesophagus^(^ ^20^ ^)^, stomach/gastric^(^ ^4^ ^)^, bladder^(^ ^24^ ^)^, oral^(^ ^20^ ^)^, larynx^(^ ^20^ ^)^ and breast^(^ ^23^ ^)^). Because we were interested in the effect of the combination of vegetables and fruit, in cases where a meta-analysis reported separate effects of vegetables only and of fruit only, we considered the lower risk estimate.

### Estimation of population-attributable fraction

The PAF represents the proportional reduction in chronic disease that would occur if all Canadians would consume the recommended number of servings of vegetables and fruit. The PAF applies the risk of inadequate vegetables and fruit consumption for chronic disease to the distribution of vegetables and fruit consumption in the Canadian population in the formula^(^ ^26^ ^)^


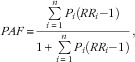

where:

*P*_*i*_ is the proportion of individuals in interval *i*;

*i* (interval) refers to the consumption of 0–<1, 1–<2, 2–<3, 3–<4, 4–<5, 5–<6, 6–<7, 7–<8 and 8+ servings/d;

*RR* is the relative risk for each serving increase in vegetables and fruit consumption;


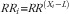
 is the relative risk for interval *i* relative to the recommended number of servings;

*X*_*i*_ is the mid value of interval *i*;

*L* is the recommended number of servings; and

*n* is the number of intervals below the recommended number of servings.

We calculated the PAF using the distribution of vegetables and fruit consumption (described above under ‘Consumption of vegetables and fruit in Canada’) and the relative risk for disease (described above under ‘Risk of inadequate vegetables and fruit consumption for chronic disease’) for each of type 2 diabetes, IHD, ischaemic stroke, and lung, colorectal, oesophagus, stomach/gastric, bladder, oral, larynx and breast cancer.

### Costs

We obtained the proportions of costs for hospital care, physician services and drugs for each of the diseases of interest from the Economic Burden of Illness in Canada (EBIC 2008) online tool^(^ ^27^ ^)^. For costs for type 2 diabetes, we further applied the proportion of 0·96 to the costs of all diabetes (type 1 and type 2 combined)^(^ ^28^ ^)^. We multiplied these proportions by the total costs (all diseases combined) in 2015 for hospital care, for physician services and for drugs, available through the Canadian National Health Expenditure Trends report^(^ ^29^ ^)^, to compute the direct health-care costs. These costs were then multiplied by the PAR to estimate the avoidable direct health-care costs. We estimated indirect costs following the human capital approach applied in the Economic Burden of Illness in Canada (EBIC 1998)^(^ ^30^ ^)^: we calculated the ratio of indirect costs to direct costs for each disease of interest, assumed these ratios to be stable over time, and used the product of the ratio and the estimated avoidable direct health-care costs for each of the diseases of interest to represent the avoidable indirect health-care costs. The economic burden represents the sum of avoidable direct health-care costs and avoidable indirect costs for all diseases of interest.

### Confidence intervals

We carried out 50 000 Monte Carlo simulations to obtain 95 % confidence intervals for the economic burden estimates. In these simulations, we assumed the reported relative risks to follow lognormal distributions and estimated variance parameters from the reported 95 % confidence intervals.

### Scenario analysis

The economic burden is an estimate of the potential cost savings if all 35·8 million Canadians were to eat adequately in terms of vegetables and fruit. As an alternative scenario, we calculated the potential cost savings for a scenario whereby all Canadians with an inadequate consumption of vegetables and fruit would increase this consumption by one extra serving daily. As a third scenario we estimated the potential cost saving of a modest 1 percentage point annual decrease in the proportion of individuals with inadequate consumption of vegetables and fruit. For this scenario, we assumed that all Canadians with inadequate vegetables and fruit consumption had an equal probability to become adequate in their consumption of vegetables and fruit. Krueger *et al*.^(^ ^17^ ^)^ had previously applied a similar scenario for decreases in physical inactivity, body weight and tobacco use, assuming the projected Canadian population from 2015 to 2035^(^ ^31^ ^)^.

## Results

Table 1 shows the distribution of vegetables and fruit consumption of Canadians. For girls below the age of 15 years, 51·3 % did not meet the recommendations of five or more daily servings of vegetables and fruit. Of these girls, 3·0, 6·0, 9·6, 15·7 and 17·0 % reportedly consumed 0–<1, 1–<2, 2–<3, 3–<4 and 4–<5 servings vegetables and fruit/d, respectively (Table 1). The prevalence of inadequate vegetables and fruit consumption ranged from 51·3 to 81·5 % by age group among girls and women and from 58·2 to 92·0 % among boys and men. For all Canadians combined, the prevalence of inadequate consumption of vegetables and fruit was 84·2 %. If all Canadians would eat the minimum recommendation, 249 million servings would be consumed daily. However, Canadians reportedly consume a total of 171 million servings daily. A total of 92 million extra daily servings of vegetables and fruit are needed to have all Canadians increase their vegetables and fruit consumption to the recommended number of servings (Table 1).

**Table 1:** Vegetables and fruit consumption in Canada by gender and age group

Gender/age	Canadian population in	Approximated recommendation	% consuming given number of servings of vegetables and fruit/d									%	Mean	Total recommended servings/d	Total consumed servings/d	Consumption deficit* servings/d
group	2015 (’000)	(servings/d)	0–<1	1–<2	2–<3	3-<4	4–<5	5–<6	6–<7	7–<8	8+	inadequate	servings/d	(’000)	(’000)	(’000)
Male																
<15 years	2948	5	2·2	8·8	14·8	17·2	15·3	11·3	9·2	5·5	15·7	58·2	5·0	14 740	14 756	3273
15–34 years	4830	8	4·2	11·4	15·6	17·4	16·8	10·2	8·0	5·7	10·7	89·3	4·6	38 640	21 992	17 820
35–54 years	4977	8	3·4	10·4	18·2	20·0	18·3	9·8	7·7	4·3	8·0	92·0	4·3	39 818	21 310	19 168
55–64 years	2402	7	2·9	10·0	20·2	22·3	16·9	9·9	6·7	4·8	6·3	88·9	4·1	16 814	9922	7278
65–74 years	1584	7	2·2	7·3	18·0	20·5	18·0	15·0	8·6	4·0	6·4	89·6	4·3	11 087	6860	4421
75+ years	1035	7	1·9	4·9	15·4	21·4	19·5	14·8	9·3	5·6	7·3	87·1	4·5	7242	4696	2679
Total	17 776		3·4	10·0	17·4	19·6	17·5	10·8	7·8	4·9	8·6	89·0	4·4	128 342	79 536	54 638
Female																
<15 years	2801	5	3·0	6·0	9·6	15·7	17·0	16·6	12·4	6·9	12·7	51·3	5·2	14 007	14 463	2538
15–34 years	4705	7	2·1	7·6	13·1	16·9	15·3	15·2	10·8	7·0	12·2	80·9	5·0	32 932	23 455	11 266
35–54 years	4973	7	1·5	5·5	11·4	16·2	18·2	16·1	11·2	8·2	11·7	80·2	5·1	34 811	25 336	11 125
55–64 years	2435	7	1·4	6·3	12·1	17·5	17·5	14·8	11·8	7·3	11·1	81·5	5·0	17 042	12 188	5638
65–74 years	1697	7	1·3	4·9	11·4	15·9	16·6	16·3	13·1	8·8	11·6	79·6	5·2	11 880	8744	3653
75+ years	1457	7	1·0	3·6	10·7	16·5	19·6	16·0	12·2	8·9	11·5	79·6	5·2	10 200	7530	3074
Total	18 068		1·7	6·1	11·9	16·6	17·1	15·6	11·5	7·7	11·8	79·5	5·1	120 873	91 716	37 294
Overall	35 844		2·5	8·0	14·6	18·1	17·3	13·2	9·7	6·3	10·2	84·2	4·7	249 214	171 252	91 933

* Consumption deficit represents the number of daily servings needed to have all Canadians with inadequate consumption of vegetables and fruit increase their consumption to the recommended number of servings.

Table 2 lists the retrieved relative risks and estimated PAF values associated with inadequate vegetables and fruit consumption for various chronic diseases by gender and age. The estimated PAF ranged from 0·9 % for colorectal cancer among girls below 15 years of age to 67·9 % for larynx and oral cancers among men aged 35–54 years. Table 2 also lists the estimated direct and indirect costs for the chronic diseases of interest by gender and age. The highest direct costs, $CAN 636 million per year, were reported for type 2 diabetes among men in the age range of 35–54 years. Table 3 reveals direct and indirect costs attributable to the inadequate consumption of vegetables and fruit by chronic disease, by age and by gender. The type 2 diabetes-associated total costs attributable to the inadequate consumption of vegetables and fruit was $CAN 789 million per year, from which 45·7 % came from direct health-care costs (Table 3). The total costs attributable to the inadequate consumption of vegetables and fruit were higher among men and higher in older age groups. Overall, the economic burden of inadequate consumption of vegetables and fruits in Canada was estimated to be $CAN 3·3 (95 % CI 0·9, 5·2) billion in 2015. About $CAN 1 (95 % CI −0·05, 1·9) billion of these were direct costs (costs for hospitals, physicians and drugs) and $CAN 2·3 (95 % CI 1·0, 3·4) billion were indirect costs due to losses in productivity (Table 3). The estimate for the economic burden was $CAN 2·3 (95 % CI 1·6, 2·9) billion, $CAN 0·6 (95 % CI 0·4, 0·8) billion in direct costs and $CAN 1·7 (95 % CI 1·2, 2·1) billion in indirect costs, when costs related to type 2 diabetes, oesophageal cancer and stomach/gastric cancer were excluded (Table 3).

**Table 2:** Relative risk (RR), population-attributable fraction (PAF) and estimated direct and indirect health-care costs associated with chronic diseases in Canada by gender and age group

					2015 costs (’000 $CAN)			
			PAF (%)		Direct†		Indirect	
Age group/chronic disease (ICD-10 code)	RR*	95 % CI	Male	Female	Male	Female	Male	Female
<15 years								
IHD (I20–I25)	0·90	0·82, 0·99	11·88	9·91	717·4	595·7	1226·3	1018·3
Ischaemic stroke (I63)	0·94	0·89, 0·99	6·94	5·74	159·3	85·2	272·3	145·7
Lung cancer (C34)	0·97	0·95, 0·98	3·40	2·79	55·4	171·2	264·4	817·6
Colorectal cancer (C20)	0·99	0·98, 1·00	1·12	0·91	30·9	34·3	147·8	163·7
Oesophagus cancer (C15)	0·94	0·88, 1·01	6·94	5·74	9·4	5·7	44·7	27·3
Stomach/gastric cancer (C16)	0·94	0·86, 1·03	6·94	5·74	8·6	19·2	40·9	91·7
Bladder cancer (C67)	0·97	0·95, 0·99	3·40	2·79	30·8	35·5	147·0	169·7
Oral cancers (C00–C14)	0·77	0·66, 0·89	29·62	25·64	1208·9	302·5	5772·3	1444·2
Larynx cancer (C32)	0·77	0·66, 0·89	29·62	25·64	34·0	36·2	162·2	172·8
Breast cancer (C50)	0·98	0·97, 1·00	2·25	1·84	10·5	18·4	50·0	87·9
Diabetes mellitus (E10–E14)	0·96	0·86, 1·07	4·56	3·76	26 914·1	13 720·2	31 926·4	16 275·3
15–34 years								
IHD (I20–I25)	0·90	0·82, 0·99	33·92	23·80	1991·7	2290·6	3404·5	3915·6
Ischaemic stroke (I63)	0·94	0·89, 0·99	21·12	14·35	5401·2	1746·6	9232·7	2985·7
Lung cancer (C34)	0·97	0·95, 0·98	10·82	7·18	640·6	962·6	3058·9	4596·4
Colorectal cancer (C20)	0·99	0·98, 1·00	3·66	2·40	2331·1	3035·6	11 131·0	14 495·3
Oesophagus cancer (C15)	0·94	0·88, 1·01	21·12	14·35	307·4	162·2	1467·7	774·4
Stomach/gastric cancer (C16)	0·94	0·86, 1·03	21·12	14·35	776·1	1277·1	3706·0	6098·2
Bladder cancer (C67)	0·97	0·95, 0·99	10·82	7·18	681·9	364·7	3255·9	1741·3
Oral cancers (C00–C14)	0·77	0·66, 0·89	67·20	52·61	1486·7	1419·3	7099·1	6777·1
Larynx cancer (C32)	0·77	0·66, 0·89	67·20	52·61	69·3	172·5	331·1	823·6
Breast cancer (C50)	0·98	0·97, 1·00	7·27	4·79	83·6	18 354·3	399·3	87 642·2
Diabetes mellitus (E10–E14)	0·96	0·86, 1·07	14·32	9·58	69 359·9	78 738·4	82 276·8	93 401·9
35–54 years								
IHD (I20–I25)	0·90	0·82, 0·99	34·80	22·39	25 681·0	16 608·2	43 898·9	28 389·8
Ischaemic stroke (I63)	0·94	0·89, 0·99	21·80	13·45	189 441·0	50 547·9	323 828·8	86 406·2
Lung cancer (C34)	0·97	0·95, 0·98	11·23	6·72	22 454·6	38 061·0	107 221·1	181 741·8
Colorectal cancer (C20)	0·99	0·98, 1·00	3·82	2·24	59 743·6	42 034·6	285 276·5	200 716·0
Oesophagus cancer (C15)	0·94	0·88, 1·01	21·80	13·45	8500·3	2267·0	40 589·0	10 824·9
Stomach/gastric cancer (C16)	0·94	0·86, 1·03	21·80	13·45	11 578·0	8056·5	55 285·1	38 469·7
Bladder cancer (C67)	0·97	0·95, 0·99	11·23	6·72	13 231·0	5777·3	63 178·3	27 586·5
Oral cancers (C00–C14)	0·77	0·66, 0·89	67·85	50·12	17 179·9	7172·7	82 034·3	34 249·9
Larynx cancer (C32)	0·77	0·66, 0·89	67·85	50·12	5277·1	1292·2	25 198·1	6170·2
Breast cancer (C50)	0·98	0·97, 1·00	7·56	4·48	849·9	218 453·9	4058·4	1 043 120·9
Diabetes mellitus (E10–E14)	0·96	0·86, 1·07	14·83	8·97	636 180·4	340 905·0	754 656·2	404 391·7
55–64 years								
IHD (I20–I25)	0·90	0·82, 0·99	28·58	23·05	49 845·1	23 924·5	85 204·8	40 896·3
Ischaemic stroke (I63)	0·94	0·89, 0·99	17·59	13·88	238 919·2	84 192·0	408 406·4	143 917·1
Lung cancer (C34)	0·97	0·95, 0·98	8·95	6·95	63 167·0	59 677·1	301 623·7	284 959·0
Colorectal cancer (C20)	0·99	0·98, 1·00	3·01	2·32	101 788·5	64 200·7	486 041·7	306 559·5
Oesophagus cancer (C15)	0·94	0·88, 1·01	17·59	13·88	19 641·4	4921·9	93 788·1	23 502·3
Stomach/gastric cancer (C16)	0·94	0·86, 1·03	17·59	13·88	16 322·4	6804·9	77 939·6	32 493·5
Bladder cancer (C67)	0·97	0·95, 0·99	8·95	6·95	31 721·7	11 669·6	151 471·4	55 722·3
Oral cancers (C00–C14)	0·77	0·66, 0·89	59·15	51·17	24 972·5	8962·2	119 243·9	42 794·8
Larynx cancer (C32)	0·77	0·66, 0·89	59·15	51·17	12 312·9	3243·8	58 794·1	15 489·4
Breast cancer (C50)	0·98	0·97, 1·00	6·00	4·63	922·0	251 645·8	4402·4	1 201 612·7
Diabetes mellitus (E10–E14)	0·96	0·86, 1·07	11·87	9·26	457 808·4	348 787·7	543 066·1	413 742·4
65–74 years								
IHD (I20–I25)	0·90	0·82, 0·99	26·70	21·69	84 631·6	59 642·5	144 668·5	101 952·3
Ischaemic stroke (I63)	0·94	0·89, 0·99	16·34	13·00	244 077·3	122 163·8	417 223·6	208 825·8
Lung cancer (C34)	0·97	0·95, 0·98	8·28	6·48	104 089·1	89 181·4	497 027·2	425 842·8
Colorectal cancer (C20)	0·99	0·98, 1·00	2·78	2·16	142 985·6	93 821·2	682 758·4	447 997·9
Oesophagus cancer (C15)	0·94	0·88, 1·01	16·34	13·00	18 510·3	5453·9	88 387·1	26 042·2
Stomach/gastric cancer (C16)	0·94	0·86, 1·03	16·34	13·00	25 162·9	13 421·4	120 153·2	64 087·6
Bladder cancer (C67)	0·97	0·95, 0·99	8·28	6·48	58 672·9	16 787·5	280 164·2	80 160·5
Oral cancers (C00–C14)	0·77	0·66, 0·89	56·40	49·01	18 600·8	9259·9	88 819·0	44 216·3
Larynx cancer (C32)	0·77	0·66, 0·89	56·40	49·01	15 704·0	3648·7	74 986·7	17 422·4
Breast cancer (C50)	0·98	0·97, 1·00	5·54	4·32	946·4	118 011·8	4519·1	563 508·5
Diabetes mellitus (E10–E14)	0·96	0·86, 1·07	10·99	8·65	405 867·9	350 053·0	481 452·7	415 243·4
75+ years								
IHD (I20–I25)	0·90	0·82, 0·99	25·09	21·21	151 947·0	220 169·9	259 736·9	376 356·5
Ischaemic stroke (I63)	0·94	0·89, 0·99	15·28	12·72	287 136·5	285 716·5	490 828·6	488 401·2
Lung cancer (C34)	0·97	0·95, 0·98	7·71	6·35	93 793·3	77 547·3	447 864·5	370 289·4
Colorectal cancer (C20)	0·99	0·98, 1·00	2·58	2·11	178 159·7	165 099·0	850 715·7	788 350·4
Oesophagus cancer (C15)	0·94	0·88, 1·01	15·28	12·72	16 483·9	6955·2	78 710·9	33 211·1
Stomach/gastric cancer (C16)	0·94	0·86, 1·03	15·28	12·72	31 140·6	24 021·6	148 696·6	114 703·6
Bladder cancer (C67)	0·97	0·95, 0·99	7·71	6·35	87 545·7	28 430·8	418 032·2	135 757·7
Oral cancers (C00–C14)	0·77	0·66, 0·89	53·94	47·92	17 833·7	11 211·9	85 156·1	53 537·0
Larynx cancer (C32)	0·77	0·66, 0·89	53·94	47·92	10 672·2	2203·7	50 960·0	10 522·5
Breast cancer (C50)	0·98	0·97, 1·00	5·15	4·23	989·2	94 321·9	4723·7	450 388·5
Diabetes mellitus (E10–E14)	0·96	0·86, 1·07	10·25	8·47	294 213·3	295 700·6	349 004·6	350 768·9

ICD-10, International Statistical Classification of Diseases and Related Health Problems, 10th Revision.

* RR per 1 serving/d increase.

† Direct costs include costs for hospital care, physician services and drugs.

**Table 3:** The economic burden of inadequate vegetables and fruit consumption by chronic disease, gender and age group in 2015 in Canada

	Attributable direct costs in 2015 (’000 $CAN)					
	All			Excluding mixed/weak evidence*		
	Direct	Indirect	Total	Direct	Indirect	Total
Chronic disease (ICD-10 code)						
IHD (I20–I25)	154 137	263481	417618	154137	263481	41718
Ischaemic stroke (I63)	239201	408888	648089	239201	408888	648089
Lung cancer (C34)	41576	198528	240105	41576	198528	240105
Colorectal cancer(C20)	22023	105161	127185	22023	105161	127185
Oesophagus cancer (C15)	13522	64570	78092			
Stomach/gastric cancer (C16)	21442	102387	123829			
Bladder cancer (C67)	20125	96095	116220	20125	96095	116220
Oral cancers (C00–C14)	66811	319022	385833	66811	319022	385833
Larynx cancer (C32)	30785	146999	177784	30785	146999	177784
Breast cancer (C50)	31638	151073	182711	31638	151073	182711
Diabetes mellitus (E10–E14)	360875	428081	788956			
Age group						
<15 years	2371	4574	6945	625	2493	3118
15–34 years	23682	42379	66061	5777	19578	25354
35–54 years	230971	468259	699230	100268	292520	392788
55–64 years	226083	543391	769474	131495	402640	534135
65–74 years	232931	563617	796548	148452	428989	577440
75+ years	286098	662066	948163	219680	543028	762708
Gender						
Male	640613	1441712	2082325	380664	1042391	1423056
Female	361523	842574	1204096	225632	646856	872488
Total	1 002 136	2 284285	3286421	606296	1689248	2295544

ICD-10, International Statistical Classification of Diseases and Related Health Problems, 10th Revision.

* Excluding Type 2 diabetes, stomach and oesophagus cancers.

**Fig. 1:**
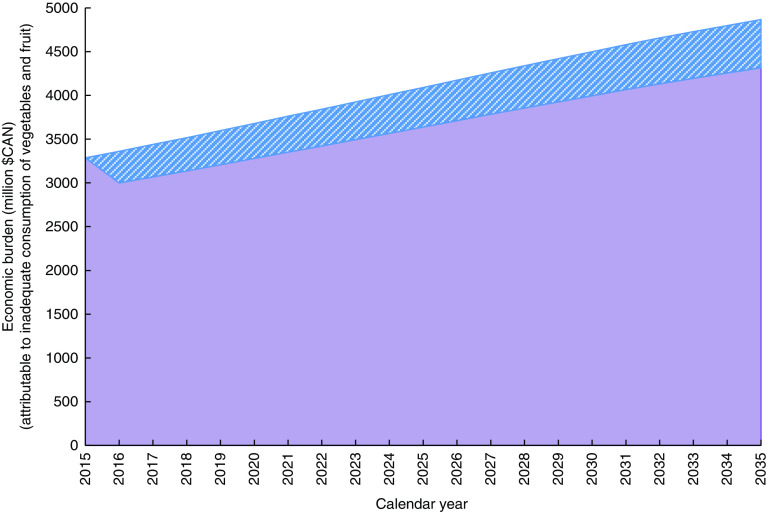
(colour online) Effect of increasing the consumption of vegetables and fruit by 1 serving/d on health care-costs in Canada: 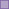, costs attributable to the inadequate consumption of vegetables and fruit; 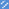, costs avoided (cumulative=$CAN 9·2 billion)

**Fig. 2:**
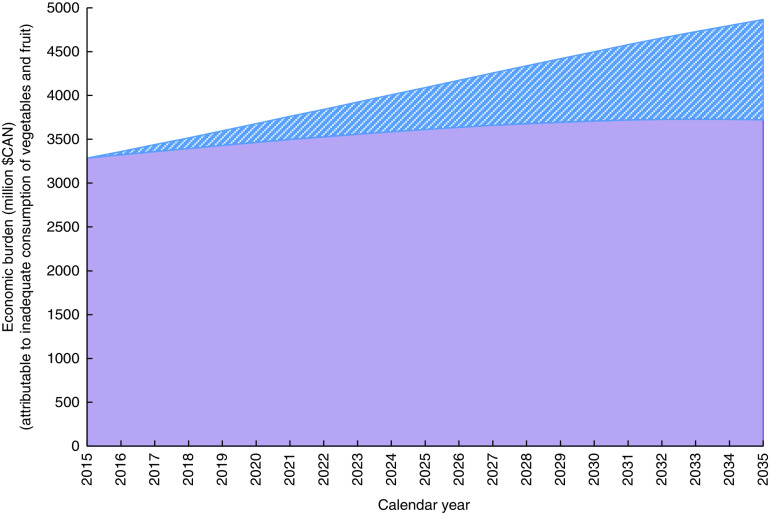
(colour online) Effect of reducing the prevalence of inadequate vegetables and fruit consumption by 1 percentage point per year on health-care costs in Canada: 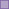, costs attributable to the inadequate consumption of vegetables and fruit; 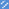, costs avoided (cumulative=$CAN 10·82 billion)

The above estimates of the economic burden represent the sum of direct and indirect costs that would be avoided if all Canadians would have diets adequate in vegetables and fruit. In a scenario where all Canadians with inadequate diets would increase their vegetables and fruit consumption by 1 serving/d, the avoided cost would total $CAN 362 million in the first year and would reach $CAN 551 million in 2035 for a total of $CAN 9·2 billion in cumulative avoided costs over 20 years (Fig. 1). In the scenario with a 1 percentage point annual reduction in the prevalence of vegetables and fruit inadequacy, avoided cost would total $CAN 39 million in the first year and would reach $CAN 1·1 billion in 2035 for a total of $CAN 10·8 billion in cumulative avoided costs over 20 years (Fig. 2).

## Discussion

The present study estimated that the cost to Canada attributable to the inadequate consumption of vegetables and fruit totals $CAN 3·3 billion per year, with 30·5 % in direct health-care costs and 69·5 % in indirect costs due to productivity losses. Canada spent an estimated $CAN 219·1 billion on direct health-care costs in 2015^(^ ^29^ ^)^, of which an estimated $CAN 19·8 billion was for the treatment and management of diabetes, CVD and cancer. About 5 % of these costs for diabetes, CVD and cancer could have been avoided if Canadians had complied with existing recommendations for vegetables and fruit^(^ ^32^ ^)^. With a Canadian population of approximately 35·8 million, the avoidable costs for health care and productivity loss approximate $CAN 92 per capita per year.

Cadilhac *et al*. estimated the economic burden attributable to a low consumption of vegetables and fruit to be $AU 269 million in 2008^(^ ^15^ ^)^, which approximates to per capita avoidable costs of $CAN 11 per Australian per year. Differences with our per capita estimate of $CAN 92 per year may originate from differences in lifestyle, culture, food choices, dietary recommendations and health-care costs between the two nations, as well as from methodological differences between the studies. With respect to the later, for example, Cadilhac *et al*. estimated avoided costs associated with a 172 g/d increase in the consumption of vegetables and fruit, whereas we estimated costs associated with inadequate consumption of vegetables and fruit using national dietary recommendations as a reference. Scarborough *et al*. estimated the costs associated with the treatment and management of diabetes, CVD and cancer attributable to poor diet to be £4·9 billion in 2006/2007^(^ ^15^ ^)^, which approximates to per capita avoidable costs of $CAN 133 per UK citizen per year. It is important to note that Scarborough *et al*. took a broader perspective by considering everyone consuming 600 g vegetables and fruit/d, having total cholesterol levels of 3·8 mmol/l and a BMI of 21 kg/m^2^ as the counterfactual of poor diet^(^ ^15^ ^)^, thus capturing more costs than those attributable to low vegetables and fruit consumption only. Because Scarborough *et al*. estimated the costs attributable to poor diet, and Cadilhac *et al*. costs associated with low vegetables and fruit consumption, the present study represents the first study to estimate costs associated with the inadequate consumption of vegetables and fruit using national dietary recommendations as the criterion for adequate consumption.

The effects of diet on chronic diseases are multifactorial and include both risk reducers and risk factors. In addition to vegetables and fruit, the WHO lists fish and fish oils, potassium and moderate alcohol intake as risk reducers for which research has provided convincing evidence, and dietary fibre, wholegrain cereals, nuts, folate and unsaturated fatty acids as risk reducers with less convincing evidence. Diets high in sodium, high in alcohol and containing *trans* and saturated fats are listed as the risk factors with convincing evidence^(^ ^33^ ^)^. Calculations that consider each of these dietary risk reducers and risk factors will produce higher estimates for the economic burden of poor diets relative to the estimates that consider solely the inadequate consumption of vegetables and fruit, the approach of the present study. Where some nations have distinct recommendations for vegetables and for fruits, Canadian recommendations specify the combined number of servings of vegetables and fruit. This poses challenges for the estimation of the economic burden. For example, in their meta-analysis, Li *et al*.^(^ ^21^ ^)^ revealed a statistically significant protective effect of fruit and of green leafy vegetables on the development of type 2 diabetes, but no statistically significant effect of the combined number of servings of vegetables and fruit on type 2 diabetes. A calculation that considers fruit and green leafy vegetables, rather than the grouping of vegetables and fruit, will provide more accurate estimates of the economic burden. However, the reality is that Canadian public health decision makers settled on a recommendation for the combination of vegetables and fruit, and thus it is desirable to provide an economic burden associated with this recommendation. Excluding type 2 diabetes, as well as oesophageal cancer and stomach/gastric cancer for which the evidence is also mixed^(^ ^34^ ^)^, revealed a substantially lower estimate, although more precise, for the economic burden ($CAN 2·3 billion per year). Some may, however, consider the latter estimate too low as it does not capture the risk-reducing effects of fruit and green leafy vegetables^(^ ^21^ ^)^.

Krueger *et al*. estimated the economic burden of smoking tobacco, excess body weight and physical inactivity to be $CAN 23·3 billion, $CAN 19 billion and $CAN 11 billion, respectively^(^ ^17^ ^)^. In their approach, Krueger *et al*. were more inclusive in their consideration of health-care costs. Specifically, where we considered costs for hospital, physician services and drugs, Krueger *et al*. also included costs for health research and for other health-care professionals (except dental) and expenditures. In 2015, the costs for items we considered constituted about 86 % of the costs for the items considered by Krueger *et al*.^(^ ^17^ ^)^. If we had been as inclusive as Krueger *et al*., our estimate for the economic burden of inadequate consumption of vegetables and fruit would have been $CAN 3·8 billion per year. The estimate of the economic burden of inadequate vegetables and fruit consumption should be considered an economic burden in addition to the estimates for smoking tobacco, excess body weight and physical activity by Krueger *et al*., because the relative risks used in our estimation originated from meta-analyses of mostly clinical trials and observational studies that were adjusted for tobacco use, excess body weight, and physical activity among other confounders^(^ ^4^ ^,^ ^20^ ^–^ ^24^ ^,^ ^28^ ^)^. By expanding the estimates by Krueger *et al*. with an estimate for the economic burden of inadequate intake of vegetables and fruit, we provide policy makers with further evidence-based options for interventions aimed at preventing chronic diseases and reducing associated health-care expenditures.

We estimated that Canadians collectively consume 171 million servings of vegetables and fruit daily and that an additional 92 million servings are needed for consumption to increase to adequate levels for all Canadians. As many vegetables and fruit in Canada can be purchased for a price of approximately $CAN 0·30^(^ ^35^ ^)^, this would translate into approximately $CAN 10 billion per year in costs to consumers. Our estimate of $CAN 3·3 billion in avoidable costs to society provides an appealing incentive for further investments in the promotion of vegetables and fruit. These investments should not only target education and behavioural change of consumers, but also strategize vegetables and fruit production. Investments and changes in the consumption of vegetables and fruit are not expected to come quick. Our scenario analyses showed that a gradual target of a modest 1 percentage point annual reduction in the proportion of individuals consuming less than the recommended servings of vegetables and fruit daily would avoid approximately $CAN 10·8 billion in 20 years and an increase of one serving of vegetables and fruit daily would avoid approximately $CAN 9·2 billion in 20 years.

Our estimates of the economic burden of inadequate vegetables and fruit consumption are based on those chronic diseases for which there are published meta-analysis showing a significant relationship with vegetables and fruit. Our estimates did not consider chronic diseases for which the relationship with vegetables and fruit has not been studied or for which there is insufficient evidence. The evidence on importance of vegetables and fruit in the causation of cancers is evolving, with recent publications suggesting a more modest risk than earlier publications. For the above reasons it is essential that our estimates be updated when new and better evidence on the health benefits of vegetables and fruit becomes available. Established limitations of dietary research apply to the present study: the assessment of dietary intake depends on self-report, which is prone to error, and reporting of food frequency does not translate accurately into absolute consumption. In addition, the CCHS summary variable on consumption of vegetables and fruit includes potatoes, which has been shown not to provide the same benefit in terms of preventing chronic disease as other non-starchy vegetables^(^ ^36^ ^,^ ^37^ ^)^. Other limitations of the present study relate to the PAF approach that did not consider a latency period, which may lead to an overestimation of the cost savings of the two intervention scenarios. As a further limitation we note that although we used direct cost estimates provided in the Canadian National Health Expenditure report^(^ ^29^ ^)^, the allocation of these costs to specific disease categories was based on the assumption that the distribution of costs by disease category and the ratio of direct to indirect costs do not change over time. However, it is likely that disease-specific costs may change due to changes in treatments over time. Third, we adapted some of the age-specific recommendations for vegetables and fruit to align them with the available age categories for health-care costs. For some age/gender groups we used the lower bound of the recommended range. Had we used a mid-range value or otherwise a higher value for the recommendation, our estimates of the economic burden would have been higher. Lastly, since the CCHS included only participants aged 12 years or older, we assumed that the distribution of vegetables and fruit consumption observed in the 12–14 years age group applied to those under 12 years of age. The overall error this may have introduced is assumed to be very small as the contribution of the ‘under 15 years age group’ is less than 0·2 % of the estimated burden (Table 3).

## Conclusion

In conclusion, we estimated that $CAN 3·3 billion of annual health-care costs in Canada are attributable to inadequate consumption of vegetables and fruit. Further investments in the promotion of vegetables and fruit will prevent chronic diseases and substantially reduce direct and indirect health-care costs.
